# Genome-scale metabolic models predict diet- and lifestyle-driven shifts of ecological interactions in the gut microbiome

**DOI:** 10.1080/19490976.2026.2694811

**Published:** 2026-07-03

**Authors:** Georgios Marinos, Karlis Arturs Moors, Kristina Schlicht, Malte Rühlemann, Silvio Waschina, Wolfgang Lieb, Andre Franke, Matthias Laudes, Mathieu Groussin, Mathilde Poyet, Christoph Kaleta, A. Samer Kadibalban

**Affiliations:** a Research Group Medical Systems Biology, Institute of Experimental Medicine, University Hospital Schleswig-Holstein Campus Kiel, Kiel University, Kiel, Schleswig-Holstein, Germany; b CAU Innovation GmbH, Kiel University, Kiel, Schleswig-Holstein, Germany; c Institute of Clinical Molecular Biology, University Hospital Schleswig-Holstein Campus Kiel, Kiel University, Kiel, Schleswig-Holstein, Germany; d Nutriinformatics, Institute of Human Nutrition and Food Science, Kiel University, Kiel, Schleswig-Holstein, Germany; e Institute of Epidemiology, Kiel, Kiel University, Kiel, Schleswig-Holstein, Germany; f Institute of Diabetes and Clinical Metabolic Research, University Hospital Schleswig-Holstein Campus Kiel, Kiel, Schleswig-Holstein, Germany; g Global Microbiome Conservancy, Institute of Clinical Molecular Biology, University Hospital Schleswig-Holstein Campus Kiel, Kiel University, Kiel, Schleswig-Holstein, Germany; h Institute of Experimental Medicine, University Hospital Schleswig-Holstein Campus Kiel, Kiel University, Kiel, Schleswig-Holstein, Germany

**Keywords:** Flux balance analysis, community modeling, ecological interactions, nutrition, microbiome, microbial ecology

## Abstract

Microbiomes and their host environments form complex, interconnected ecosystems. The microbial species within a microbiome, on the one hand, compete for resources, while on the other hand, they exchange vital metabolites to support their survival. These interactions are influenced by the microbial genetic repertoire, environmental conditions, and availability of nutrients. We developed EcoGS (http://www.github.com/KaletaLab/EcoGS), a metabolic modeling tool designed to predict the ecological interactions between pairs of microbes. Applying EcoGS to the microbiomes of two distinct human cohorts revealed a shift from collaborative to exploitative ecological interactions associated with increased dietary intake of simple sugars (glucose and fructose) in diabetic individuals and those living industrialized lifestyles. On the other hand, the consumption of cobalamin (vitamin B12), phylloquinone (vitamin K1), and biotin (vitamin B7), among other compounds, was associated with increased collaboration in the gut microbiome. We conclude that the abundance of simple sugars as an energy source reduces the necessity for microbes to cooperate, thereby increasing competition and hostility among microbiome members. Moreover, our study proposes multiple compounds, such as urate, deoxyadenosine, deoxyguanosine, and hypoxanthine, for in vitro validation tests as dietary interventions that have the potential to restore the ecological balance within the community. EcoGS serves as a valuable tool for exploring microbiome dynamics and their connections to environmental changes and disease.

## 1. Introduction

The interactions among microbes, as well as between microbes and their host, are complex, yet understanding these relationships is critical for elucidating how the microbiome influences human health. The gut microbiome plays a key role in a variety of diseases, including inflammatory conditions such as inflammatory bowel disease, where current therapies primarily target inflammation rather than directly modulating the microbial community.^1^ It also contributes to metabolic disorders, including obesity and diabetes, and influences host immune function and behavior through the gut‒brain axis.^2^ Like all microbial communities, the trillions of microbes in the human gut do not act in isolation; they form intricate ecological networks that mediate cooperation, competition, and metabolic exchange. Although each individual's microbiome is unique, external factors such as lifestyle and environment significantly shape community composition and function.^3^ These considerations highlight the importance of investigating microbiome structure and function, as well as the diversity and stability of microbial ecological networks, using advanced computational tools.

Ecological theory considers that microorganisms interact with each other and their environment, resulting in different growth profiles for individual microorganisms in comparison to their growth in isolation. Those interactions can be exploitative (amensalism, competition, and antagonism), collaborative (commensalism and mutualism), or neutral.^4^ To study those interactions, a basic approach is to model pairs of two species without taking into account the effect of other species. To this end, the co-growth capabilities of two species are directly associated with their ability to compete or collaborate for resources. Specifically, their co-growth rates should be lower than their single individual growth rates in the case of competition, more so in the case of collaboration, and the same in the absence of interaction.^5^

To understand interactions within the microbiome, it is important to study the co-occurrence of the involved microbes. Metabolically dissimilar species are more likely to co-occur, as they do not compete for the same resources. Furthermore, metabolic competition is positively associated with phylogenetic relatedness.^6^ ^,^ ^7^ However, metabolic cooperation can still be predicted. Specifically, cases of close cooperation loops among species, where the products of one species are a nutritional source for other species, have been predicted in communities.^5^ Interestingly, cooperation can lead to the stability of communities in the case of nutritional perturbations, while competition ensures stability in the case of invasive species occurring.^6^ Beyond species co-occurrence, it is also important to consider how functions are distributed across community members and how they are interdependent, as these features further shape the relationship between community structure and function. For example, it was shown that metabolic functional redundancy could increase in IBD microbial communities despite their lower taxonomic diversity.^8^

While the above-mentioned ecological concepts appear to be theoretically straightforward, inferring them for the gut microbiome is challenging. Importantly, this is because a vast number of microbes cannot be cultivated,^9^ and the physicochemical environment of the gut that strongly influences microbiome structure is highly variable.^10^ Moreover, the human gut microbiome is influenced by human lifestyle and environmental factors, as urbanization has put pressure on species that were favored by the pre-agricultural way of life.^3^ To this end, a seminal review on the effect of nutritional groups (e.g., amino acids, fats, carbohydrates) and dietary styles (e.g., Western diet) on the abundance of species was published.^11^ For instance, the lack of microbiota-accessible carbohydrates in Western-style diets is a reason for species extinction in the gut, which otherwise could produce short-chain fatty acids, leading to detrimental effects, such as dysbiosis, for the host.^12^ Likewise, recent work on the infant gut microbiome has shown that human milk oligosaccharides, as microbial substrates, can shape cross-feeding interactions and broader competitive and cooperative relationships within the gut ecosystem.^13^

It is important and computationally possible to predict the presence or absence of microorganisms and, therefore, the structure of the microbiome. Useful tools such as MetaBIDx can improve species prediction in metagenomes.^14^ Nonetheless, there is a need for tools that allow the categorization of ecological interactions within the microbiome as a prerequisite for a targeted manipulation of microbiome structure and function.^15^ One key approach that allows for computationally predicting ecological interactions within microbial communities is by simulating the metabolic capabilities and growth potential of the respective species. To this end, constraint-based modeling builds on genome-scale metabolic reconstructions of the metabolic networks of microbial species that can be derived using automatic tools, such as gapseq.^16^ Therefore, apart from the genome of the species, nutritional cues, which serve as a proxy for lifestyle and environment, can also be taken into account. Such models can be used for simulating the growth profiles of individual microbial species based on linear programming approaches. Flux balance analysis (FBA) is an example of this approach. Briefly, FBA determines the flux (i.e., input, output, and internal reaction rates) through a metabolic network that maximizes a specified objective function, such as cellular growth. This calculation incorporates the stoichiometric structure of the metabolic network of a simulated organism and relevant environmental constraints, including nutrient availability.^17^ Furthermore, it is possible to simulate complex microbial communities using approaches such as community FBA. This technique merges multiple metabolic models into one, which can then be used in FBA simulations. In those simulations, it is possible to compare and contrast the growth of species alone and their growth when they are co-occurring. The co-growth profiles are calculated based on the exchanges of compounds between the models and the availability of common compounds. In an exploitative interaction, the uptake reactions of the models compete for the same bounds, reducing the species' feasible capabilities (i.e., solution space), which leads to less growth of the individual species. In collaborative interactions, the production of compounds from one species boosts the growth capability of the other, expanding the solution space. FBA can capture those interactions for each compound, providing a readout of the co-growth profiles for each model. In this way, ecological interactions emerge naturally from the model without adding any assumptions beyond the shared environment and metabolic capabilities.

Metabolic modeling has enabled the prediction of ecological interactions between host and microbes,^18^ can be used for the design of pre- and probiotics^19^ and can explore the effect of dietary patterns on the host based on whole-body metabolic models.^20^ The importance of this field is not limited to human-associated systems but also extended to others (e.g., in chickens.^21^ Advancements in the available community metabolic modeling software and tools have been reviewed in the literature.^22^ ^,^ ^23^ MICOM^24^ and MicrobiomeGS2^25^ are examples of software that implement community metabolic modeling without taking into account a structured spatiotemporal environment (which is the case for BacArena^26^ and COMETS.^27^ The main practical difference of MicrobiomeGS2 in comparison with MICOM is that MICOM uses a two-step procedure that balances between individual and community-level growth rates. Besides, MicrobiomeGS2 is implemented in R, while MICOM is available in Python. MicrobiomeGS2 has been used by other studies for reconstructing personalized community microbial models of humans^28^ and nematodes.^29^

To predict ecological interactions within microbial communities, we present EcoGS, a user-friendly extension of MicrobiomeGS2 that infers pairwise microbial interactions under defined nutritional conditions from genome-scale metabolic models. The software accepts SBML metabolic models produced by gapseq^16^ and loaded with sybil^30^ as input and is implemented as an R package available on GitHub (https://github.com/KaletaLab/EcoGS). In contrast to existing constraint-based frameworks such as MICOM, MicrobiomeGS2, and COMETS, which focus primarily on community growth or metabolite fluxes, EcoGS systematically classifies interactions into exploitative, collaborative, or neutral categories based on relative growth outcomes and summarizes them as abundance-weighted ecological profiles at the community level. This design enables scalable and interpretable comparisons across individuals, cohorts, and environmental contexts without requiring spatiotemporal simulations or optimization of complex community objectives. We applied EcoGS to the gut microbiomes from two human cohorts to investigate how diet, disease, and lifestyle, including industrialized versus non-industrialized settings, shape microbial ecological interaction structures.

## 2. Methods

### 2.1. Cohort data

In this study, data from two independent cohorts were analyzed. The PopGen cohort comprises three subcohorts (FoCus, BSP, and SPC) totaling 2396 individuals and encompassing comprehensive dietary data, 16S rRNA gene sequences of the gut microbiome, and clinical data (PopGen Biobank, Schleswig-Holstein, Germany). The metadata variables included age, gender, body mass index (BMI), smoking status, diabetes, inflammatory bowel disease (IBD), and other relevant health parameters. The cohort consisted of 1329 females and 1067 males, with 274 individuals having diabetes (Supplementary Figure S1). The data of the 108 individuals diagnosed with IBD were filtered out before the downstream analysis, as IBD has a substantial influence on the gut microbiome structure and dynamics.^31^ To examin the temporal stability of EIRs and weighted ecological interaction frequencies, we used data of a Spanish longitudinal cohort of T2D patients that were previously recruited at the Hospital Universitari Dr Josep Trueta (Girona, Spain), the cohort data included genomic DNA^32^ and species count data that were previously produced.^28^ Patients received either metformin or a placebo and were followed up at 2 months (M2) and 4 months (M4). For this study, a subset of placebo patients (*N* = 18) was used to model EIRs. For demonstration purposes, closely adjacent timepoints (Baseline vs Month 2 and Month 2 vs Month 4) were pooled. The 16S rRNA sequences (V1–V2 regions) of the gut microbiome of PopGen were mapped against a reference database of 822 human gut microbial genomes derived from the AGORA resource^33^ using USEARCH version v11,^34^ identity threshold = 97%, and query minimum coverage = 95% using the following command:

usearch -usearch_local query.fasta -db agora.fasta -strand both -id 0.97 -blast6out mapped.csv -maxaccepts 1000000 -maxrejects 1000000 -query_cov 0.95.

The AGORA gut bacterial genomes cover more than 800 representative species; it can be considered a trustworthy reference database. We used the full extent of the database; therefore, we did not take into consideration any phenotypic information in our selection. The mapping identified 409 microbial strains with varying abundances across the cohort. The second cohort in this study comprised 1229 participants as part of the Global Microbiome Conservancy (GMbC) initiative.^35-37^ This cohort consisted of 789 individuals from non-industrialized communities and 440 from industrialized communities (Supplementary Figure S2). In this study, we used metagenomic whole-genome assemblies from the GMbC cohort for downstream simulations, so no mapping against reference genomes was needed. The metadata of the GMbC cohort included age, gender, and body mass index (BMI).

### 2.2. Creating and simulating the community models

gapseq (version 1.2)^16^ was used to create genome-scale metabolic models for both the reference (AGORA) microbial genomes and the metagenomic bins of the GMbC cohort, utilizing a standardized North German diet.^28^ This diet imposed uptake limits (i.e., lower bounds) for the respective exchange reactions of nutritional compounds, enabling the practically unlimited secretion capabilities (i.e., upper bounds) of each reaction. Thereafter, the biomass reaction of the metabolic models was optimized based on flux balance analysis (see introduction on the topic) using the R^38^ package *sybil,* ^30^ and the achieved baseline growth rate was recorded. Subsequently, the models were paired using the MicrobiomeGS2 R package. Since the models were built on the same software, the naming conventions, reactions, and metabolite IDs were automatically compatible. The exchange reactions of each submodel were connected to a common extracellular space, so that the flux through the submodel was possible. After joining each pair of models, and to simulate the community growth, the objective coefficients of each sub-model were set to one; the objective coefficients of the other reactions were set to 0. In the next step, following a published approach, we set the following coupling constraints: c = 400 mmol.g_Dw_^−1^.h^−1^ as originally proposed^39^ and a more relaxed coupling u = 1^−6^ mmol.g_Dw_^−1^.h^−1^. Then, we performed flux balance analysis, as originally implemented in MicrobiomeGS2. We explicitly recorded the exchange reactions whose fluxes predict the exchange of compounds among the sub-models when one flux is positive and the other is negative, respectively. To exclude numerical artifacts, the prerequisite for reporting was that the respective absolute fluxes were bigger than 1^−6^. To further ensure the quality of the results, we recorded each case if both submodels could grow (cut-off of 1^−6^) and if each optimization process was successful. Both the simulations of the stand-alone models and the joined models were conducted using the academic version of the linear solver IBM ILOG CPLEX through its R interface, cplexAPI,^40^ and using the Sybil method hybbaropt. Both simulations run in parallel using multi-core computing based on PSOCK clusters in R.

### 2.3. Inference of the ecological interactions

**Figure 1.**
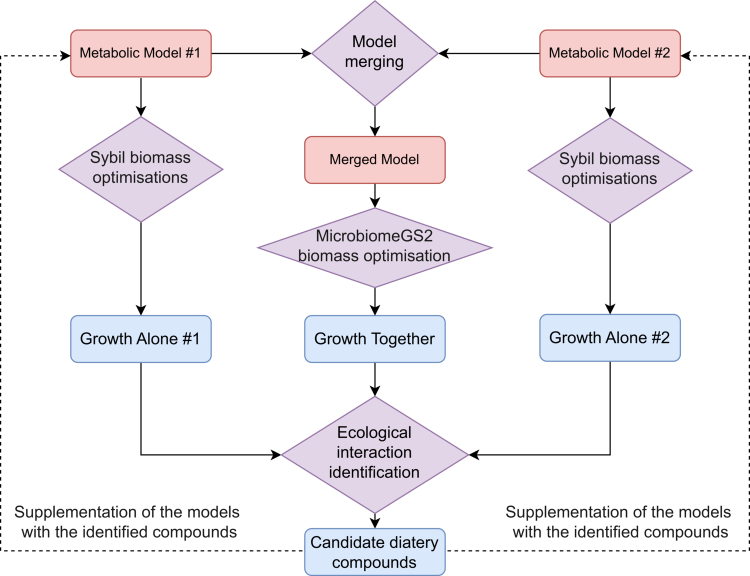
Diagram representation of the EcoGS pipeline: red boxes represent metabolic model data, blue boxes represent predictions extracted from the models, purple diamond shapes represent operations and functions included in the package, continuous arrows represent the data flow between each step, and dashed arrows represent the feedback of candidate intervention data for predicting their influence.

For each of the 409 microbial species found in participants in the PopGen cohort and the 483 species found in participants of the GMbC cohort, we compared the predicted single-growth rate (SG) with its co-growth rate (CG) when joined with each of the other microbial species belonging to the same cohort (Figure 1). A change in co-growth rate was considered if it differed from the single-growth rate by a tolerance interval margin of at least 5% (i.e., | $\frac{\text{SG}\;-\;\text{CG}}{\text{SG}}|>0.05$ ) (Table 1). The extent of change in growth is not taken into account (treated equally) as long as it exceeds the tolerance interval (margin). For instance, a change of 10% or 100% in the growth is equally considered beneficial. The 5% cutoff was selected as a small but biologically meaningful effect size that exceeds the numerical variation inherent to linear programming solvers. However, to verify the effect of our threshold selection, we additionally repeated the analysis across thresholds from 0% to 10% (0, 0.1, 0.5, 1, 2, 5, 7, 10%) (see Supplementary Figure S3). Based on these comparisons, we constructed a symmetrical matrix representing the inferred ecological interactions between species pairs for each of the two cohorts, encompassing a total of 83,436 pairs for the PopGen microbes (Supplementary Table S1) and 116,403 pairs for the GMbC microbes (Supplementary Table S1). The number of pairs can be calculated as follows:

$$\mathrm{Number}\;\mathrm{of}\;\mathrm{combinations}=\frac{n\times \;(n-1)}{2},$$

n : the number of microbes in the cohort.

**Table 1.** Definition of the ecological interactions for two species, a and b.

Model #a growth comparison	Model #a interaction effect	Model #b growth comparison	Model #b interaction effect	Ecological interaction	Interaction category
CG_a_ > SG_a_ + m	Benefit	CG_b_ > SG_b_ + m	Benefit	Mutualism	Collaborative
SG_a_ − m < = CG_a_ > = SG_a_ + m	No change	CG_b_ > SG_b_ + m	Benefit	Commensalism	Collaborative
SG_a_ − m < = CG_a_ > = SG_a_ + m	No change	AG_b_ − m < = CG_b_ > = SG_b_ + m	No change	Neutralism	Neutral
CG_a_ > SG_a_ + m	Benefit	CG_b_ < SG_b_ − m	Harm	Antagonism	Exploitative
SG_a_ − m < = CG_a_ > = SG_a_ + m	No change	CG_b_ < SG_b_ − m	Harm	Amensalism	Exploitative
CG_a_ < SG_a_ − m	Harm	CG_b_ < SG_b_ − m	Harm	Competition	Exploitative

CG: co-growth, SG: single growth, m: margin, in this study = (single growth)*5%.

Next, we categorized each microbial species pair into one of the six possible ecological interactions (Table 1, Figure 1). Thereafter, we counted the number of pairs belonging to each ecological interaction in each microbial community (4236 communities in the two cohorts combined) and weighted those counts by the abundance of the species in each pair.

Let the abundances of species S1, S2, S3, S4, S5, etc. be denoted as A1, A2, A3, A4, A5, etc. For a given ecological interaction type *E*, the total number of species pairs exhibiting this interaction in the community is NE​, represented as (S1​, S2​), (S1​, S3​), (S3​, S5​), etc. EcoGS offers a weighing for the ecological interaction, W_NE_​​, in two different ways depending on the context:

$${W1}_{\mathrm{NE}}=(A1\times A2)+(A1\times A3)+(A3\times A5)+\;\ldots$$

$${W2}_{\mathrm{NE}}=\;\min(A1,A2)+\;\min(A1,A3)+\;\min(A3,A5)+\;\ldots$$

W1_NE_ takes into account the probability of two species encountering each other in the community as the multiplication of their abundances. W2_NE_ takes into account that the limiting factor in metabolic exchange between two species is the minimum abundance of the two species. While the first weighing method (W1_NE_) is appropriate in the context where the probability of the species interaction is relevant, such as in in vitro culture, here, we opted to use the second approach (W2_NE_), as the biologically relevant limiting factor for the metabolic exchange between two species in the gut environment is the lower abundance of the two species.

The ecological interaction network of a microbiome has a mesh topology with single-type nodes, where every node (species) is connected to all other nodes with no hubs. The edges represent the predicted ecological interaction between the two nodes they connect, with the edge weight calculated as described above. The ecological interaction ratios (EIRs) are inferred from the ecological interaction network and represented in a table, where each row corresponds to one microbiome. The EIRs for one microbiome are calculated from the ecological interaction network by summing up the weights for one interaction type and dividing them by the summed weights of another, and transforming them with log10. To address the inherent codependency in compositional data, a logarithmic transformation of the ratio between pairs of variables is often applied.^41^ In this study, the proportions of different ecological interactions are compositional data. In the context of the six microbial ecological interactions, this approach yielded 15 log-transformed ecological interaction ratios (EIRs), such as *log(antagonism/competition)*, *log(commensalism/mutualism)*, and so forth.

The log-transformed proportional data of the ecological interaction ratios per microbial community (with pseudoanonymized IDs) are in Supplementary Table S1 for the PopGen cohort and in Supplementary Table S1 for the GMbC cohort.

### 2.4. Phylogenetic inference of the GMbC species

Phylogenetic trees were reconstructed using SGB representative genomes with the identify, align, and infer functions of GTDB-Tk v2.3.2 and GTDB Release R07-RS207.^42^ ^,^ ^43^ The bacterial and archaeal trees were rooted with representatives of Patescibacteria and Nanoarchaeota, respectively, and joined at the root to yield a combined phylogenetic tree encompassing both domains.

### 2.5. Dietary interventions

To predict the effects of dietary interventions on the ecological interactions within the gut microbiome of the PopGen cohort, simulations were performed for the supplementation of 105 individual nutrients (metabolites). Each simulation involved modifying the microbial diet in *gapseq* models by adjusting the lower bounds of the exchange reaction for the respective metabolite. The supplementation amount for each nutrient was set to two times the value of the standardized North German diet (Supplementary Table S1). For each intervention, the ecological interaction prediction pipeline was applied to microbial pairs independently, generating 105 ecological interaction matrices with the same dimensions and structure as the original pre-intervention matrix. Thereafter, post-intervention ecological interaction ratios per participant were calculated.

### 2.6. Statistical analysis

For the PopGen cohort, linear models (LMs) were employed to assess associations between the ratios of ecological interactions (EIRs) and four key clinical variables: age, body mass index (BMI), gender, and smoking behavior (measured as cigarettes per day), as well as with diabetes. The model structure was specified as follows:

lm(EIR~BMI+age+gender+diabetes+smoking).

Additionally, EIRs were tested for associations with individual dietary metabolites, which were inferred from dietary questionnaires. Each metabolite was analyzed separately, adjusting for the same four clinical covariates:

lm(EIR~dietarymetabolite+BMI+age+gender+diabetes+smoking).

Similarly, for the GMbC cohort, linear models were applied to examine the associations between EIRs and age, BMI, gender, and lifestyle (classified as industrialized or non-industrialized):

lm(EIR~lifestyle+age+BMI+gender).

Following the metabolic supplementation simulations, the effect of each supplementation on the EIRs was evaluated by comparing the EIRs before and after supplementation using a paired Wilcoxon signed-rank test. Finally, the phylogenetic distances between each pair of microbes in the GMbC cohort were compared across the six ecological interaction categories using pairwise Wilcoxon signed-rank tests. A false discovery rate (FDR)^44^ correction was applied to the resulting *p*-values to account for multiple testing after each statistical test. Adjusted *p*-values less than *α* = 0.05 were considered statistically significant.

### 2.7. Software

The community model simulations were conducted in a high-performance computing environment in an R environment (version 4.4.1).^38^ Specifically, the following R packages were used: MicrobiomeGS2^25^ version 0.2.0, cplexAPI^40^ version 1.4.0 (along with the IBM ILOG CPLEX Optimization Studio/Academic Initiative version 22.10), *sybil* ^30^ version 2.2.1, doParallel^45^ version 1.0.17, stringr version 1.5.1,^46^ lattice version 0.22-6, Matrix version 1.7-1, iterators version 1.0.1 4, data.table version 1.17.8, and foreach^47^ version 1.5.2.

All statistical analyses were conducted with R (version 4.5.0) within the RStudio environment.^48^ Plots were created with the ggplot2 R package (version 3.5.2)^49^ and R base plotting functions. The results of all the statistical tests were determined to be significant below a threshold of *α* = 0.05. *p*-values were adjusted for multiple testing using FDR.^44^

## 3. Results

### 3.1. Ecological interaction and metabolic exchange among microbial communities and across phylogeny

**Figure 2.**
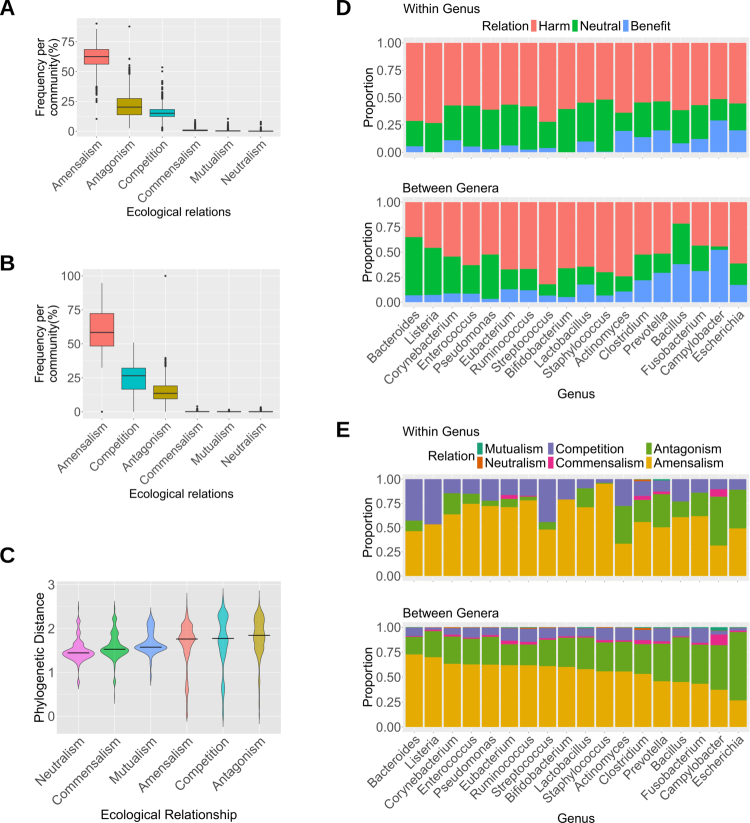
Ecological interactions among microbial communities. (A) The frequencies of the six types of ecological interactions among the gut microbiome of the PopGen cohort; the x-axis is ordered by the median of the ecological interaction frequencies across all communities. (B) Similar to A. for the GmbC cohort microbial communities, the colors correspond to those in A. (C) A violin plot representing the distribution of the phylogenetic distances between pairs of microbes among the six different types of ecological interactions for microbes found in the GMbC cohort participants. The black line in the middle of each box represents the median value. The violins are sorted by the median on the x-axis. The colors correspond to those in A. and B. (D) Bar plots depicting the interaction outcome for the 18 most abundant genera in the PopGen cohort. The top part shows the outcome of interactions with species from different genera. The bottom part shows the outcome of interactions with species belonging to the same genus. The genera are ordered on the X-axis to correspond to the order in E. (E) Bar plots depicting the ecological interaction proportions for the 18 most abundant genera in the PopGen cohort. The top part shows the ecological interactions between microbes of the corresponding genus and different genera. The bottom part shows the ecological interactions between species belonging to the same genus. The genera are ordered on the X-axis in correspondence with their frequency of amensalistic interaction with other genera.

While ecological interaction categories (amensalism, competition, antagonism, commensalism, mutualism, and neutralism) address the relationship between the interacting species, it is also noteworthy to examine the interaction effect on each model (microbial species) independently (harmful, beneficial, and neutral). The ecological interactions among the gut microbial communities of both cohorts had considerably higher proportions and higher variances of exploitative ecological interactions in comparison to the collaborative and neutral interactions. The PopGen cohort has more antagonism than competition, while the GMbC cohort has more competition than antagonism. The two cohorts have similar relative frequencies of commensalism, mutualism, and neutralism (Figure 2A and B). Furthermore, species pairs with exploitative interactions tend to have higher phylogenetic distances with bimodality, suggesting that very closely related species also tend to have exploitative interactions, while collaborative and neutral interactions tend to have lower to intermediate phylogenetic distances, as observed in the GMbC cohort (Figure 2C). In order to evaluate the differences in the ecological interactions between different microbial genera, the 18 most abundant genera in the gut communities of the PopGen cohort participants were selected.

The interaction effect of each model was estimated within each genus and between genera. The former takes into account the interactions among microbial species belonging to each of the genera, and the latter between the species of each of the genera and the species of all other genera. (Figure 2D). Similarly, the proportion of the ecological interactions (considering the interaction effect on both microbes in the pair) was estimated for microbial pairs within each genus and between genera (Figure 2E).

The proportions of interaction effects, on each model, vary between the different genera; the interactions among species from the same genus produce more neutral and less beneficial effects than interactions with species from different genera. The ecological interaction categories also vary between different genera. Higher antagonism, lower competition, and higher commensalism are observed in interactions between species belonging to different genera in comparison to interactions within the same genus.

Not only does *Escherichia* have the highest frequency of antagonistic interactions in comparison with other genera, but also, antagonism comprises the highest proportion of all types of ecological interactions between *Escherichia* and other genera. Moreover, those antagonistic interactions are mostly harmful to *Escherichia* (Figure 2D and E and supplementary Table S2). *Campylobacter, Clostridium*, and *Eubacterium,* on the other hand, have the highest proportions of collaborative interactions with other genera and within the same genus. Whereas *Fusobacterium*, *Prevotella*, *Ruminococcus*, and *Eubacterium* have relatively high competition against other genera (Figure 2E and Supplementary Table S2). On the other hand, *Bacteroides* species showed a highly amensalistic trend between genera and a competitive trend within the genus. *Staphylococcus* exhibits a very high proportion of amensalistic interactions within the same genus.

**Figure 3.**
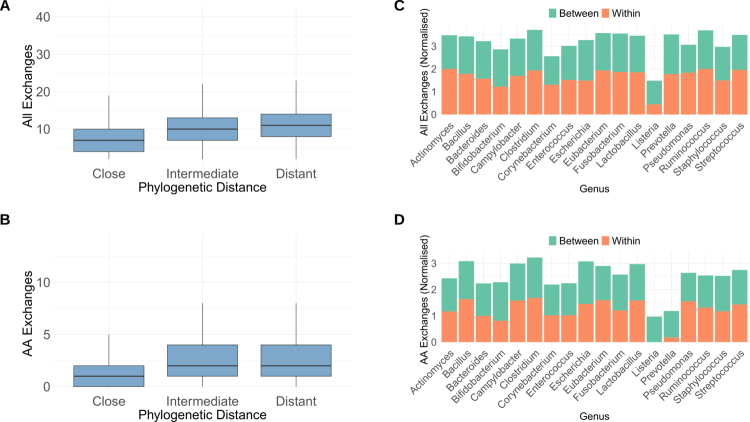
The number of metabolic exchanges between microbial pairs across phylogeny and genera. (A) The number of predicted exchanged metabolites between microbial pairs on the Y-axis across three binned categories of phylogenetic distance between the microbes in each pair on the X-axis. The phylogenetic distance binning was set based on the distribution of the bimodal phylogenetic distances (Figure S4) as follows: close: distance < = 0.8, intermediate: 0.8 < distance < = 1.3, and distant: distance > 1.3. (B) Same as A, but only counting the number of amino acid exchanges between the microbes in the pairs. (C) A bar plot of the normalized rates of predicted metabolic exchanges between microbes in pairs within the same genus in orange and those belonging to different genera in green. (D) Same as C., but only counting the normalized rates of amino acid exchanges between the microbes in the pairs.

Furthermore, we observed a positive association between the number of predicted metabolic exchanges among microbial pairs in the GMbC cohort and their phylogenetic distance using a Spearman test (*p*-value < 2.2^−16^, rho 0.33); this relationship remained significant even when considering only amino acid exchanges (*p*-value < 2.2^−16^, rho 0.17) (Figure 3A and B). In contrast, in the PopGen cohort, when comparing exchange frequencies within the same genus versus across different genera, after normalizing by the number of interacting pairs in each category, we found no strong differences in either total metabolic exchanges or amino acid-specific exchanges. The only notable exception was *Listeria* and *Prevotella*, which exhibited substantially more amino acid exchanges within their respective genera than other genera did (Figure 3C and D).

Additionally, to assess how differences in community diversity influence ecological interaction profiles, we tested the association between multiple alpha-diversity measures (Shannon index, Simpson's diversity, and richness) and ecological interaction ratios (EIRs) in the PopGen, longitudinal (Spanish), and GMbC cohorts. Across all the diversity metrics examined, higher alpha diversity was consistently associated with an increase in collaborative and neutral relative to exploitative interactions and an increase in mutualism relative to neutralism (Supplementary Table S2). These associations were significant after false discovery rate correction, where detected. Importantly, no alpha-diversity measure was associated with a shift toward increased exploitative interactions.

To assess the temporal stability of predicted ecological interaction profiles, we compared within-individual to between-individual multivariate distances in the Placebo group of the Spanish cohort (measured at months 0, 2, and 4; corresponding to visits V1, V3, and V5). For each individual, we computed pairwise Euclidean distances across either the 15 ecological interaction ratios (EIRs) or the six weighted ecological interaction frequencies. Weighted frequencies were z-scored prior to distance computation to prevent high-magnitude interaction types (e.g., amensalism) from dominating the distance metric. Between-individual distances were computed from randomly sampled pairs of samples belonging to different individuals.

We evaluated adjacent intervals only (V1–V3 and V3–V5 pooled) for EIRs and weighted frequencies. Across both data types, within-individual distances were significantly smaller than between-individual distances (Wilcoxon rank-sum test, all *p* < 0.001, Supplementary Figure S4). For EIRs, the mean within-individual distance was 0.517 (adjacent), compared to a stable between-individual mean of ~0.85. For weighted frequencies, the within-individual mean was 1.824 (adjacent), against a between-individual mean of 3.20. We observed that interindividual variation in the EIRs and weighted ecological frequencies, measured by Euclidean distance, was significantly higher than the intraindividual variance. The temporal stability of our ecological signatures also remains consistent when (1) comparing only baseline vs M2: EIR Within mean = 0.483, between mean = 0.858, *p* < 0.001; (2) comparing only M2 vs M4: EIR Within mean = 0.553, between mean = 0.813, *p* < 0.001; and even when (3) comparing baseline vs M4: EIR Within mean = 0.561, between mean = 0.870, *p* < 0.001. Moreover, we found multiple negative correlations between the longitudinal change in alpha diversity (Δdiversity between baseline and M4) and the change in multiple interaction ratios (ΔEIRs between baseline and M4) of the exploitative/collaborative types (supplementary Table S2), which corresponds to the observed associations between the alpha diversity and relative increase in collaborative interaction.

### 3.2. Associations between phenotypic and dietary data and the ecological interaction ratios

**Figure 4.**
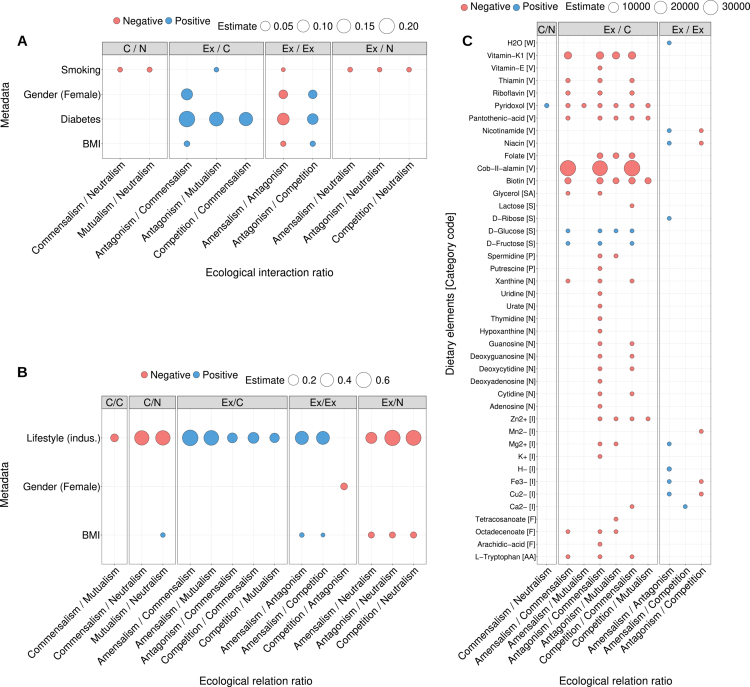
Associations between phenotypic and dietary data with ecological relation ratios. Interaction ratios are grouped by types of transitions between collaborative (C), exploitative (Ex), and neutral (N) interactions. (A) Associations between the metadata of the PopGen cohort participants and the ecological interaction ratios in their gut microbiomes. Each dot represents one significant association resulting from (LM) between four phenotypes (metadata) on the Y-axis and the ecological interaction ratios on the X-axis (log transformed). The color represents the directionality of the association, blue for positive and red for negative associations. The size of the dots depicts the LM estimate (coefficient of correlation). The interaction ratios are divided into three panels to distinguish between three types of interaction ratios: exploitative–exploitative, exploitative–collaborative, and collaborative–collaborative. To enhance readability, we grouped the compounds into categories (W for water, V for vitamin, SA for sugar alcohol, S for sugar, P for polyamines, N for nitrogen-containing compounds, I for ions, F for fatty acids, and AA for amino acids). (B) Associations between dietary elements from the diet of the PopGen cohort (y-axis) and the estimated ecological relation ratios of their gut microbiomes (x-axis). The color and size of the dots and the two panels are the same as those in A (there are only two panels because there are no significant associations with the collaborative–collaborative type of interaction ratios). (C) Associations between metadata of the GMbC cohort participants (x-axis) and the ecological interaction ratios in their gut microbiomes (y-axis). The color and size of the dots and the two panels are the same as those in A.

The linear regression models revealed significant associations between the ecological interaction ratios (EIRs) and phenotypical data from both cohorts (e.g., a positive association between diabetes and the proportion of antagonism/mutualism, *p*-value = 0.007, estimate = 0.15, and antagonism/commensalism, *p*-value = 0.0003, estimate = 0.21, Figure 4A and Table S2). Such a positive estimate reflects an increase in the numerator (antagonism) relative to the denominators (mutualism and commensalism). Similarly, a negative estimate reflects a decrease in the numerator relative to the denominator (e.g., lifestyle vs. antagonism/neutralism, *p*-value = 2.36^−22^, estimate = −0.6, Figure 4B and Table S2).

In the PopGen cohort, the antagonism proportion relative to commensalism and mutualism was significantly increased in the gut microbiome of female participants in comparison to males (*p*-values: 0.048, 0.61, respectively, supplementary Table S2). However, we did not observe the same effect in the GMbC cohort (Figure 4A and B). Smoking, on the other hand, was associated with higher neutral interactions relative to collaborative and exploitative ones (*p*-values in supplementary Table S2) and higher antagonism relative to mutualism, *p*-value = 0.025 (Figure 4A). A positive association between BMI and the proportion of antagonism relative to commensalism was observed in the PopGen cohort (*p*-value = 0.042). However, in the GMbC cohort, we only observe a decrease in exploitative interactions relative to neutralism in association with BMI (*p*-values: neutralism vs. amensalism = 0.003, neutralism vs. competition = 0.0006, neutralism vs. antagonism = 0.0006) (Figure 4A and B). Moreover, not only is there a relative increase in antagonism relative to collaborative interactions associated with diabetes (*p*-values: antagonism vs. commensalism = 0.0003, antagonism vs. mutualism = 0.007), BMI (*p*-values: antagonism vs. commensalism = 0.04), smoking (*p*-values: antagonism vs. mutualism = 0.025) and in females (*p*-values: antagonism vs. commensalism = 0.048), but antagonism is also increased relative to the two other exploitative interactions (amensalism and competition) in association with the same four phenotypical data types (Figure 4 and supplementary Table S2). Furthermore, we observed an increase in amensalism, competition, and antagonism at the expense of collaborative ecological interactions in individuals living in industrialized communities in comparison to individuals living non-industrialized lifestyles in the GMbC cohort (*p*-values: amensalism vs. commensalism = 2.83^−26^, amensalism vs. mutualism = 1.62^−16^, competition vs. commensalism = 1.91^−05^, competition vs. mutualism = 0.01, antagonism vs. commensalism = 0.0006). However, the proportions of exploitative and collaborative interactions relative to neutral interactions are significantly lower in industrialized populations (*p*-values: amensalism vs. neutralism = 0.001, competition vs. neutralism = 2.72^−17^, antagonism vs. neutralism = 2.36^−22^, commensalism vs. neutralism = 3.00^−54^, mutualism vs. neutralism = 2.59^−48^) (Figure 4B and supplementary Table S2).

Furthermore, we tested for associations between EIRs and different nutrients from the diet of the PopGen cohort. Thirty-two of the tested dietary elements showed a negative association with at least one exploitative relative to collaborative ecological interaction types, including vitamins, ions, fatty acids, nucleotides, polyamines, as well as the amino acid tryptophan (Figure 4C and supplementary Table S2). The majority of those dietary elements had significant associations with increased commensalism. The strongest associations were observed in cobalamin (vitamin B12) (amensalism vs. commensalism *p*-value = 0.026 and estimate = −37,576.7, competition vs. commensalism *p*-value = 0.024 and estimate = −39,403.4, and antagonism vs. commensalism *p*-value = 0.014 and estimate = −33,915), as well as phylloquinone (vitamin K1) and biotin (vitamin B7), which have reduced exploitative relative to collaborative interaction ratios (*p*-values and estimates in supplementary Table S2). On the other hand, we observed a significant increase in all three exploitative interactions relative to the commensalism associated with higher dietary glucose and fructose intake, as well as an increase in antagonism relative to mutualism in association with glucose intake (Figure 4C and supplementary Table S2).

### 3.3. Dietary interventions to alter the ecological interaction distributions along gut microbiomes

To identify dietary components that could alter the ecological interaction ratios and restore the observed shifts associated with diseases such as diabetes, we simulated metabolic interventions of individual metabolites; thereafter, we assessed the impact of the simulated individual nutrients on the predicted interaction ratios (see Methods). Among the tested metabolites, 19 showed a significant effect on these ratios. Supplementation with certain compounds, including the fatty acid octadecanoate, the nucleotide cytidine, seven purines (deoxyadenosine, guanosine, xanthine, urate, deoxyguanosine, hypoxanthine, and adenosine), and two vitamins (thiamin B1 and riboflavin B2), was associated with an increase in collaborative interactions (mutualism and commensalism) relative to exploitative interactions (antagonism and competition).

**Figure 5.**
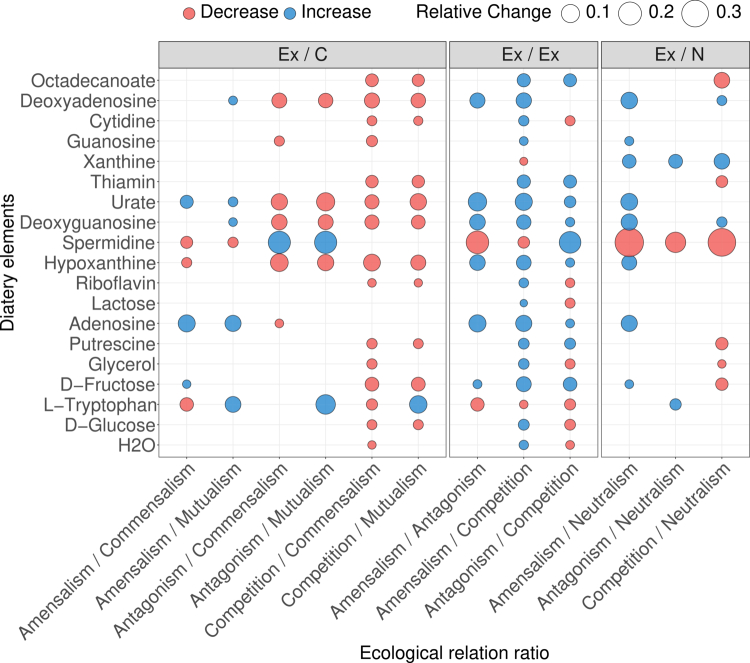
The effect of dietary metabolic intervention (Y-axis) on the ecological relation ratios (X-axis). Interaction ratios are grouped by types of transitions between collaborative (C), exploitative (Ex), and neutral (N) interactions. The dots represent the significant difference between the interaction ratios with and without the intervention, as detected with the Wilcoxon signed-rank test. The color of the dots represents the direction of the change caused by the intervention, red for a decrease in the interaction ratio, and blue for an increase. The size of the dots shows the fold change caused by an intervention, depicted as the median of all relative changes among all communities. The two panels discriminate between exploitative-collaborative and exploitative-exploitative interaction ratios.

Notably, supplementation with D-glucose and D-fructose increased collaborative interactions, whereas L-tryptophan supplementation increased exploitative interactions (Figures 4C and 5; see Discussion).

Within the exploitative category, several interventions altered internal ratios, with most increasing amensalism relative to antagonism and competition, and in some cases, increasing antagonism relative to competition. Notably, L-tryptophan produced the opposite pattern. Additional shifts were observed between exploitative and neutral interactions. Some metabolites promoted amensalism over neutralism, some promoted neutralism over competition, and only spermidine increased neutralism relative to all three exploitative interaction types (Figure 5).

## 4. Discussion

In this study, we introduced *EcoGS*, an open-source R-based computational tool designed to infer ecological interactions among microbial species from genome-scale metabolic models. By simulating the growth of each species both alone and together with another, *EcoGS* predicts interaction types: exploitative interactions (amensalism, antagonism, and competition), collaborative interactions (commensalism and mutualism), and neutral interactions, which are summarized at the community level. This framework offers a scalable and user-friendly approach for exploring microbial ecology in complex communities and is freely available as an R package (www.github.com/KaletaLab/EcoGS).

Applying *EcoGS* to gut microbiomes from two distinct human cohorts, the PopGen cohort from Germany and the GMbC cohort encompassing both industrialized and non-industrialized populations, yielded key insights into how host-related metabolic factors, lifestyle, and diet shape microbial interaction networks. Notably, in both cohorts, we observe higher proportions of exploitative interactions than collaborative ones (Figure 2A and B). This pattern is in line with the previously observed frequent co-occurrence among competing species, suggesting that shared habitat filtering drives microbiome structure.^7^ It also aligns with controlled experiments showing that competitive and amensal interactions dominate in synthetic human gut communities (comprising ~68% of all pairwise interactions, vs. ~5% cooperative ones),^15^ as well as with recent data showing a predominance of negative interactions in natural gut isolates.^50^

The phylogenetic distance of interacting species revealed more exploitative interactions between distant species (Figure 2C). However, we also observed a bimodal distribution of pairwise distances among microbes engaged in exploitative interactions, suggesting that those interactions also occur among closely related and distantly related taxa (Figure 2C). In addition, a positive correlation between the number of metabolic exchanges and phylogenetic distance (Figure 3A and B) supports the idea that closer species are metabolically similar and share similar enzyme systems, carbon source preferences, and oxygen requirements. Owing to this niche overlap and evolutionary constraints on metabolic complementation, they tend to engage in less frequent metabolite sharing.^51^

Our observations on the association between alpha diversity measures and ecological interaction ratios indicate that more diverse microbiomes tend to exhibit more cooperative and neutral ecological structures, whereas lower-diversity communities are enriched for exploitative dynamics. Since reduced microbiome diversity is a common feature of many microbiome-associated diseases, such as inflammatory bowel disease (IBD),^52^ this shift toward exploitative interactions may represent an underlying ecological mechanism linking diversity loss to microbiome dysfunction. Together, these findings suggest that altered ecological interaction structures may mediate the functional consequences of reduced microbiome diversity in destabilized microbial communities.

In the PopGen cohort, we observed an increase in antagonism relative to commensalism in individuals with a higher body mass index (BMI) and in individuals with diabetes, as well as an increase in antagonism relative to mutualism in diabetic individuals. This shift away from collaborative or neutral interactions may reflect a disrupted or less cooperative microbiome in metabolically compromised states, potentially reinforcing disease-associated dysbiosis. However, in the GMbC cohort, there was only an increase in neutralism over other types of interactions in association with BMI. This could be due to the structure of the cohort, which includes individuals with different lifestyles. With this, our study extends findings from a previous study^53^ across independent populations. In that previous study, it was found that elevated blood glucose levels and the presence of diabetes were associated with a marked increase in exploitative microbial interactions, particularly antagonism. Additionally, the analysis of the 5-y follow-up data revealed remarkable nuances in the composition of the subjects' microbiome as well as in the interaction types among the species. Species richness declined, whereas community evenness increased, indicating a shift toward more balanced abundance distributions among the remaining taxa. These trends might be the result of age- or lifestyle-dependent changes in host physiology that selectively reduce rare community members and thus enable dominant species to achieve more equitable representation. Despite these temporal variations, individual signatures were still distinguishable over time, as within-individual shifts were substantially lower than between-individual variation. Still, the amount of temporal drift we observed is a reminder that repeated sampling strengthens any longitudinal study design.

Moreover, in the GMbC cohort, microbiomes from participants with industrialized lifestyles displayed a dual trend: a rise in exploitative interactions over collaborative ones, alongside an increase in neutral interactions. This suggests that industrialization not only tilts microbial communities toward more competitive dynamics but also introduces ecological stasis or tolerance, where species coexist without significant interaction, a possible reflection of ecological simplification driven by uniform diets, reduced microbial diversity, or environmental exposures associated with industrialization.^54^ Together, these findings indicate that both metabolic disease and industrialization converge on a common ecological footprint: reduced cooperation and increased exploitation within the gut microbial community.

Both diabetes and industrialization often imply higher availability of simple carbohydrates, highlighting a potential mechanism by which modern diets contribute to microbiome instability and drive metabolic dysregulation, as the availability of simple, rapidly metabolized energy sources reduces the need for microbial cooperation.

In the human gut, it is suggested that species have distinct roles, from hydrolyzing complex carbohydrates to primary and secondary fermenters that produce short-chain fatty acids and species that use hydrogen for their metabolism.^55^ However, simple sugars such as glucose are universally possible to assimilate and energetically cheap to metabolize, which makes them an evolutionarily preferred carbon source when available.^56^ When such substrates are abundant, most community members can independently access energy without relying on complementary metabolic pathways provided by other microbes. This leads to a breakdown in mutualistic and commensal interactions, as cross-feeding and metabolic interdependence become less advantageous. As a result, community dynamics shift toward individualism, with species competing more directly for the same resources, thereby fostering exploitative interactions and reducing ecological cohesion. A consistent driver across these interaction shifts is their association with nutrient intake, particularly simple sugars. In the PopGen cohort, the consumption of glucose and fructose was associated with an increase in exploitative interactions, suggesting that these rapidly metabolized substrates may amplify microbial competition or antagonism for shared resources. In contrast, the consumption of vitamins, nitrogen-containing compounds, polyamines, and tryptophan promoted collaborative interactions, particularly commensalism. This is in line with the notion that those compounds are expensive to build up or must be consumed by the environment as public goods.^55^

Together, our findings highlight a consistent ecological signal across disease, lifestyle, and diet, which is why dietary modulation may offer a strategy for restoring cooperative microbial interactions in dysbiotic or industrialized microbiomes. However, modeling outcomes depend on microbiome structure and the definition of nutritional constraints; higher-order ecological events cannot be easily captured.

Nevertheless, the intervention analysis highlights that targeted nutrient supplementation can modulate the ecological interaction structure of the gut microbiome, but these effects are not always aligned with observational associations from dietary intake. For example, while higher habitual intake of simple sugars (glucose and fructose) was linked to increased exploitative interactions in cohort analyses, supplementation with these same sugars in silico increased collaborative interactions. This apparent contradiction likely reflects fundamental differences between dietary patterns and supplementation scenarios in a controlled metabolic model. In the real world, sustained high intake of simple sugars may reduce resource diversity and increase competition for easily accessible substrates, promoting exploitative dynamics. In contrast, supplementation in a model context can create an abundance of a readily metabolizable carbon source for both species, reducing competitive pressure. This “dose” dependency, in turn, increases their predicted growth rates and is later interpreted as an increase in collaboration. Additionally, metabolic modeling assumes a well-mixed, host-independent system and evaluates pairwise interactions in isolation. Under such conditions, removing the competition for carbon sources would promote co-growth. In vivo, spatial structure, nutrient filtering, and host regulation constrain these effects. Thus, the divergence between cohort-level and simulated results likely arises from both contextual differences (chronic vs. acute exposure) and model simplifications, emphasizing that metabolic modeling-based intervention analysis should be followed up with experimental validations.

Vitamins and purines consistently promoted collaborative dynamics in silico, supporting the view that micronutrients and nucleotides act as public goods within microbial communities.^57^ ^,^ ^58^ These compounds often require specialized biosynthetic pathways, so supplementation may relieve metabolic bottlenecks and encourage positive-sum exchanges, reinforcing cooperative interactions. Besides, we observe that nitrogen-containing compounds (cytidine, deoxyadenosine, guanosine, xanthine, urate, deoxyguanosine, hypoxanthine, and adenosine) are boosting collaborative interactions. This is particularly interesting if we consider that the colon, except towards its end, is usually a nitrogen-depleted environment.^55^

Collectively, these findings emphasize that nutrient availability strongly governs microbial interaction patterns, but the effect direction can depend on the abundance context, metabolic accessibility, and whether resources are universally utilizable or pathway-specific. This suggests that dietary interventions aimed at restoring microbial cooperation must consider not only nutrient type but also the systemic context and metabolic redundancy within the community.

Currently, EcoGS models only pairwise interactions, as the textbook definition involves two species. Therefore, our approach does not take into account the complexity of the communities. Although higher-order modeling is theoretically possible, the combinatorial explosion, e.g., ~89 million triplet interactions for all strains in the AGORA gut collection, renders it computationally infeasible with current approaches. However, ignoring multi-species interactions may underestimate cooperative cross-feeding, which requires more than two partners, or overstate competitive exclusion in cases where a third species mitigates conflicts. This is why future approaches should focus on approximating higher-order effects through iterative simulations for minimal microbial communities. An additional limitation concerns the underlying data used for metabolic reconstruction. For the PopGen cohort, the microbial community composition was inferred from 16S rRNA sequencing, which provides limited taxonomic resolution and assumes representative metabolic potential based on reference genomes. This approach may not fully capture strain-level functional variability or horizontal gene transfer, potentially limiting the accuracy of model-based interaction predictions. In contrast, the GMbC cohort was profiled using metagenomic sequencing, specifically metagenome-assembled genomes, which enabled a more direct genome-scale reconstruction. Importantly, our analyses were performed within each cohort independently rather than across cohorts, minimizing the impact of methodological differences and batch effects on the interpretation. Moreover, precision diet modeling is limited by the need to re-run simulations for each specific diet, which is computationally intensive. Additionally, the reliance on gap-filling algorithms during genome-scale reconstruction via gapseq may reduce the number of unique metabolic exchanges, biasing interaction classifications toward competition. Future studies should evaluate the tradeoff of using non-gap-filled draft models for predicting ecological interactions. Furthermore, in our present study, we assumed that species could use their entire metabolic network, while in reality, pathway activity is regulated depending on intrinsic and environmental cues. Such information could, in principle, be integrated through contextualization of metabolic networks with (meta-)transcriptomic or (meta-)proteomic data.^59^ However, those known sources of modeling uncertainty, including gap-filling and 16S-based metabolic inference, are unlikely to systematically bias interaction classifications in a direction that aligns with specific host phenotypes or dietary variables. Instead, such limitations are expected to introduce stochastic error across interaction types. The consistent associations observed across independent cohorts and dietary perturbations, therefore, argue against these patterns being driven solely by systematic modeling artifacts. Besides, while these limitations could affect the accuracy of individual pairwise predictions, the aim of this study was to capture relative ecological patterns at the community level. The most experimentally tractable predictions are the nutrient-dependent transitions between exploitative and collaborative interactions, which can be tested in controlled co-culture systems such as bioreactors using culturable gut isolates. Although cultivating gut microbes can be a challenging task,^60^ future work combining genome-resolved metagenomics and targeted experimental validation will be essential to quantify prediction accuracy at the level of individual interactions.

Moreover, the results of the longitudinal analysis show a consistent and significant separation of within-individual and between-individual distances, indicating that ecological interaction profiles retain individual-specific signatures over the study period. In summary, EcoGS provides a scalable and interpretable framework for investigating microbial ecological interactions across human populations, dietary patterns, and health states. It integrates a reproducible workflow to predict, classify, and validate pairwise ecological interactions from community FBA outputs, a layer of analysis not offered by existing tools. By linking shifts in cooperation and exploitation to diet, lifestyle, and metabolic health, EcoGS generates testable predictions that can guide in vitro validation or the design of precision-nutrition strategies aimed at restoring stabilizing community structures. Future extensions, including multi-species modeling, dynamic nutrient flux integration, and tighter empirical coupling, will further enhance its utility for both ecological theory and microbiome-based interventions. Overall, EcoGS contributes conceptually and practically to microbiome systems biology, bridging metabolic modeling, ecological theory, and translational applications.

## Supplementary Material

Supplementary Table S2.xlsx Supplementary Table S2.xlsx

Supplementary Table S1.xlsx Supplementary Table S1.xlsx

Supplementary Material Supplementary Figures.docx

## Data Availability

‐ Samples and data for the PopGen cohort were provided by the PopGen Biobank (Schleswig-Holstein, Germany) and can be accessed via a structured application procedure (https://www.uksh.de/p2n/Information+for+Researchers.html). Upon application, the PopGen data can be obtained within 7–30 d. ‐ The GMbC participant data and metadata can be requested through the Global Microbiome Conservancy website (https://microbiomeconservancy.org/). All data and metadata are distributed via controlled-access systems in accordance with the original informed consent provisions. Access to data and metadata requires a formal application and a data-access agreement. ‐ The statistical analysis and plotting R scripts and data can be found under: https://doi.org/10.6084/m9.figshare.30149005.

## List of abbreviations

EcoGS: The presented tool to predict the **Eco**logical interactions based on **G**enome-**s**cale metabolic models
GMbC: **G**lobal **M**icro**b**iome **C**onservancy
EIR: In this document, it refers to the **E**cological **I**nteraction **R**atios
FDR: **F**alse **D**iscovery **R**ate, a method for correcting for multiple testing
BMI: **B**ody **M**ass **I**ndex, calculated by dividing the weight by the height squared
