# Supplementary material for: Genome-scale metabolic models predict diet- and lifestyle-driven shifts of ecological interactions in the gut microbiome
Source: Gut Microbes. 2026 Jul 3;18(1):2694811. doi: 10.1080/19490976.2026.2694811 (PMC13336287; doi:10.1080/19490976.2026.2694811)
Supplement: Supplementary Material — Supplementary Figures.docx [file KGMI_A_2694811_SM9814.docx]

# Supplementary material

**Figure S1: The distribution of PopGen cohort descriptive phenotypic data**


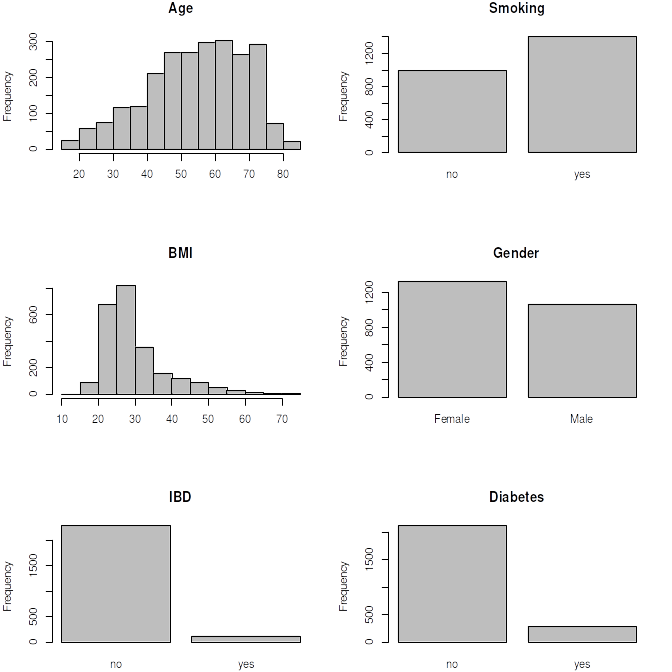


**Figure S2: The distribution of GMbC cohort descriptive phenotypic data**


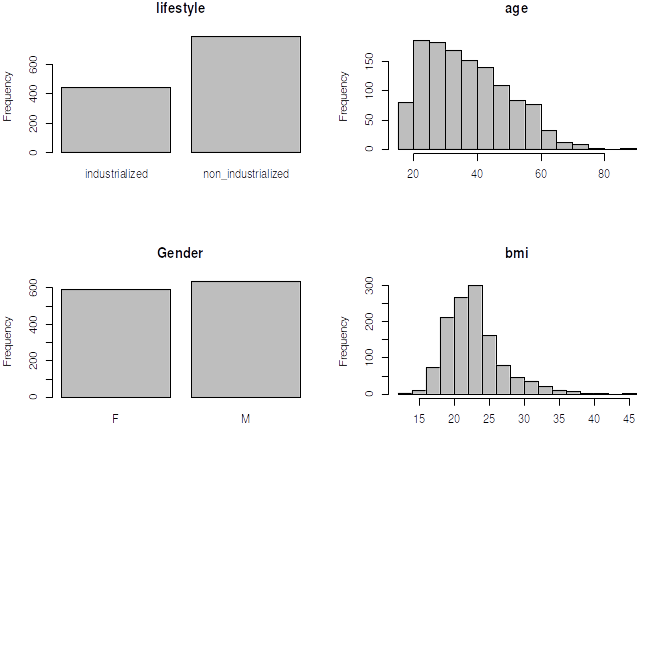


#

**Figure S3: The distribution of the phylogenetic distance among bacterial pairs of the GmbC cohort**

**
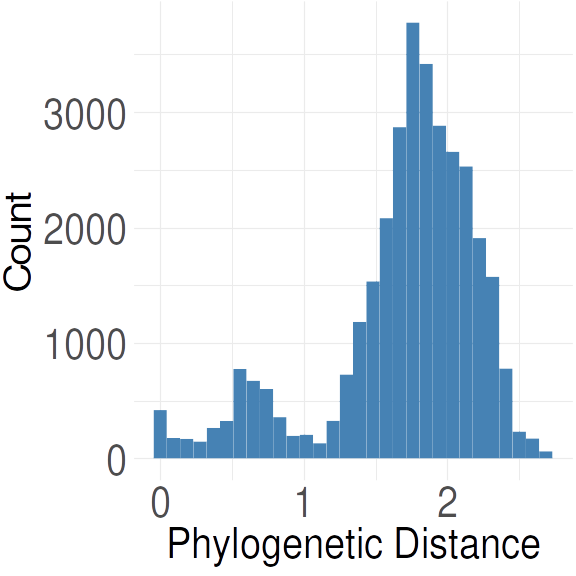
**

**Figure S4:** **The temporal stability of the ecological interaction ratios (EIRs) and ecological interaction frequencies.**

#


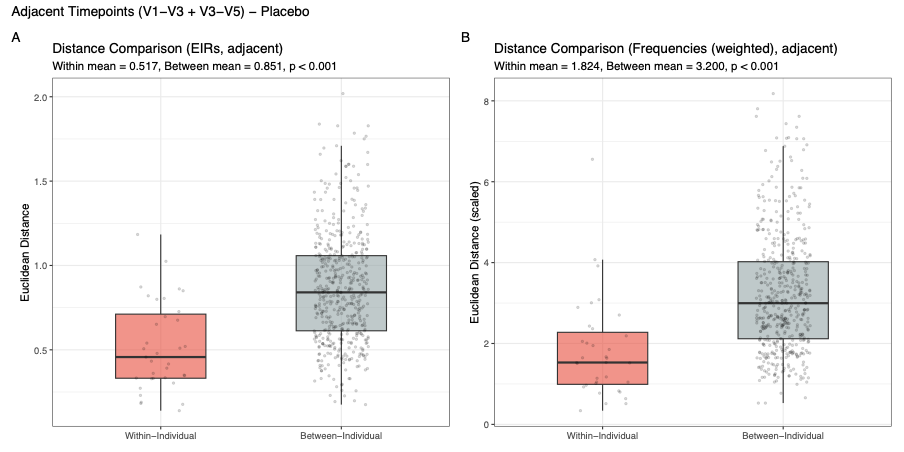


Within- versus between-individual distances at adjacent timepoints (V1–V3 and V3–V5 pooled) in the Placebo group. (A) Euclidean distances computed across 15 ecological interaction ratios (EIRs). (B) Euclidean distances computed across 6 z-scored weighted ecological interaction frequencies. In both cases, within-individual distances were significantly lower than between-individual distances (Wilcoxon rank-sum test, p < 0.001), demonstrating that ecological interaction profiles retain individual-specific signatures over short time intervals. Points represent individual distance values.

**Figure S9: Stability**
